# The State of Lunacy in England

**Published:** 1860-10-01

**Authors:** 


### Art. VI.—THE STATE OF LUNACY IN ENGLAND.

The Fourteenth Report of the Commissioners in Lunacy, although not lacking in interest, does not, from the multiplicity of its details, readily admit of a satisfactory analysis. The Commissioners first describe the additions made in 1859 to the amount of public accommodation in asylums for pauper lunatics, and the chief improvements effected therein. The new asylum for the united counties of Beds, Herts, Hants, and the borough of Bedford, it is stated, would be opened in the course of the year; and it is to be presumed that the asylum is now in use. The asylum is placed upon the most elevated portion of an estate, consisting of 260 acres; and from the building, which is a cheerful-looking and handsome structure of white brick relieved with red, extensive views are obtained in every direction. The general plan is spoken of as good, and the arrangement of the various wards convenient. The asylum is built for 500 patients, at an estimated cost of 65,000*l.*

Certain additions which have been made to the Chester Asylum during the past year receive special attention from the Commissioners. They express the opinion that the plans adopted (copies of which, together with a detailed description, &c., are given in the Appendix to the Report) will "afford valuable assistance, in the way of suggestion, to committees of visitors and their architects, upon whom the duty may hereafter devolve, or be now under consideration, of providing additional accommodation in asylums for the pauper lunatics of counties and boroughs."

The alterations and additions proposed or suggested in other of the older asylums are also reported.

An account is, moreover, given of the new asylum for the county of Northumberland, opened for the reception of patients in March, 1859. The following are a few of the particulars:—

"The asylum is designed to accommodate 210 patients, exclusive of twenty-two beds in the infirmaries, and is erected upon an elevated site, commanding an extensive view, situate at Cottingwood, about three-quarters of a mile distant from the town of Morpeth. The site is reduced to a perfectly level surface, and the subsoil, which is chiefly gravel, sand, and clay, is thoroughly drained by rubble and tile drains. The entire estate contains ninety-nine acres of land.

"The building is of brick, with stone dressings; the foundations are of rubble, masonry and concrete; the external walls are built with a two-inch void, to prevent the transmission of warmth and damp; pressed bricks are used, which give sufficiently smooth surfaces to the walls, as to require no plastering to the interior of the wards.

"The staircases in the wards are all constructed of stone in short flights, and Fox and Barrett's patent fire-proof principle, combined with brick arching, is adopted throughout the ground-floor story in all the wards. The floors are all boarded, and the ground-floor is protected from cold and damp by pugging placed beneath the floor-boards.

"The window-sashes of the single sleeping-rooms and associated dormitories on the ground-floor are of cast iron, made to slide upon brass sheaves, leaving unglazed spaces of one pane in breath when open; those of the associated dormitories on the first floor are of wood, with iron sash-bars moulded to match; the whole of the windows are made to open to a safe point only, and are secured by key latches. . . . . .

"The whole of the wards are warmed by open fires only. The ventilation is effected by two foul-air shafts in the towers, placed at the convergence of the three wards on each side of the building, with which the single sleeping-rooms, associated dormitories, and all other rooms and water-closets, communicate by large trunks in the roofs, flues formed in the walls, and apertures near the ceilings; all the flues and trunks are regulated by dampers or valves, the warm fresh air is admitted to the rooms, &c., near the floor; the shafts are provided with boilers and hot-water cisterns which serve to supply the baths, slop-rooms, &c., with hot water, as well as to promote the extraction of the foul air. . . . .

"The asylum consists of a main building, two stories in height, and two other wards of one story high detached from, but communicating with the main building by covered passages; the work-shops, laundry, offices, &c., are contained in two separate blocks. The medical superintendent's residence, surgery, and chapel are situated at the centre of the main building on its south front; and on the north side of the centre, the principal road of approach being on that side, are the residence and office of the clerk and steward, the matron, servants' rooms, general stores, and offices, and the general kitchen, which communicates with the several wards separately by covered ways; and at each extremity of the main building are the infirmaries.

"The wards in the main building extend east and west from the centre, and are occupied by the male and female patients respectively; each side is divided into three wards for different classes of patients.

"The ground floors of the wards contain ambulatories, single sleeping rooms, associated dormitories, attendants' rooms, day or dining rooms, &c. The first floors, which are occupied only at night, contain associated dormitories (facing the south), attendants' rooms, lavatories, &c.

"Each side of the building is provided with bath-rooms, containing warm and shower baths, which are conveniently situated to and common to the several wards." (p. 5.)

The Sussex Asylum, after considerable delay, occasioned by unexpected engineering difficulties encountered in procuring water, was opened in July, 1859. A description of this asylum is not given, but it is stated that the land belonging to it is about 120 acres in extent, twelve acres being under cultivation as kitchen-garden. The estimated number of patients for whom accommodation is provided is 425—viz., 212 males, and 213 females.

The vexed question of an asylum for the city of London would appear to be at length satisfactorily settled. The Commissioners state that they are glad to report that there is every prospect of an asylum being erected, without further delay, near Dartford, for 300 patients.

Two lunatic hospitals were opened in the course of 1859—one at Gloucester, and the other at Nottingham.

The Gloucester hospital (Barnwood House, formerly a gentleman's residence, now considerably altered and enlarged to meet the requirements of a hospital) affords accommodation for about 35 patients of each sex. The land attached to the house amounts to 48 acres, and is well covered with timber, a considerable portion adjoining the buildings being laid out as gardens and shrubberies.

The arrangements are very satisfactory, and on the property there are several cottages, the largest of which will be appropriated to the use of one or two ladies.

The Nottingham Hospital is an entirely new structure, built and furnished at a cost of about 18,500*l*. The site is elevated, and distant about two miles from the city, and about 17 acres of land are attached to the building. The following extracts from the architect's description will be found of interest:—

"The building consists of a centre block, containing the residence of the officials in the front and the recreation-room and kitchens at the back, with the wings for the patients towards the east and west, the whole presenting a frontage to the south of 270 feet. The galleries, which run east and west, open to the private rooms on the north, and the associated rooms on the south. The latter being arranged into four blocks, projecting at intervals from the galleries, are enabled to have windows on two or more sides of the rooms, while sufficient space is left for windows in the south wall of the galleries.

"The large associated day-rooms and dormitories are built with detached chimney stacks running up in the centre of the rooms, forming blocks of about six feet by five feet, but pierced in the centre with arched openings, ten feet high and three feet wide. In each of the large day-rooms the lower part of this aperture is filled by two open fire-grates placed back to back, the open space between them forming a warm-air chamber, the whole being covered down with an iron slab, faced with ornamental tiles, and at a height of three feet from the floor. Ranging with the smoke-flues on either side the fire-grates are flues for fresh and foul air, communicating with inlet and outlet apertures, and by means of these and the warm-air chamber before named, an effective system of ventilation is established in both day-rooms and dormitories, and a stream of warm air is supplied to the latter. The outer half-brick walls of these flues are built with Rufford's white glazed bricks, and present a clean, bright surface.

"The patients being enabled, as it were, to form a double circle round the fire, and to see each other through the arched opening above the fire-grates, it renders this arrangement of the places more conducive to the cheerfulness of the apartment, and in regard to heat, it certainly is more economical than if they were placed, as they otherwise must be, against the outer wall." (p. 16.)

This combined system of warming and ventilation is applied, though in a different manner, to the rest of the building. The amount of accommodation for patients is not stated.

The Commissioners illustrate the progress of improvement in the management of lunatic hospitals and licensed houses by a detailed account of the changes made from time to time during the past ten years, in the management and care of patients in St. Luke's Hospital and Hoxton House. The particulars form an interesting contribution to the history of the amelioration of the condition of the insane in this country.

The Commissioners announce that, as respects the metropolitan district, they have practically come to the resolution "not to add to the number of licensed houses, unless for special reasons applicable to the particular case." For example: "In the event of a medical man or other person of high character and qualifications, and possessing adequate pecuniary resources, applying for a licence to receive private patients in a suitable house, we should be disposed to make an exception, but should in that case generally, if not invariably, limit the licence to patients of one sex."

The Commissioners further write:—

"The licensed houses within our immediate jurisdiction, judging from the actual numbers of patients resident therein, appear fully to meet, not merely the requirements of the special locality (which would be comparatively unimportant, inasmuch as private patients are, for the most part, sent to asylums not in the neighbourhood of their homes), but in general the wants of the community. We have also to observe, that, in consequence of the now rapid withdrawal of the pauper patients from the five large metropolitan houses at present licensed to receive that class of the insane, extensive provision will shortly be made for the accommodation of patients of the middle and poorer classes, for whom it is hoped that ultimately adequate means of care and treatment will be afforded in public hospitals." (p. 19.)

The reception of pauper patients into private asylums is discouraged by the Commissioners as much as possible; and from the recent opening of several new county asylums, and the enlargement of others, they have been enabled to prohibit generally the reception, in the metropolitan houses, of pauper patients from distant localities; moreover, they have "lately made the licences of these houses subject to the condition that, unless upon special grounds, and with our written permission, no pauper patients shall be admitted, excepting from the counties of Middlesex and Surrey, or from places within seven miles of London."

The Commissioners next glance at the evils connected with the transfer of licences, and then proceed to a detailed report of the condition of the different private asylums in the Metropolitan district. These will be read with no small interest by those who have been affected by the recent popular, and indeed official, outcry against private asylums. Of the five large metropolitan licensed houses in which pauper lunatics are received, the Commissioners sum up their account by stating, "that as respects treatment and general comfort, they are now approximating to a very satisfactory condition." Of the houses for the reception of private patients only, the accounts are so entirely inconsistent, perhaps with one doubtful exception, with the sweeping official assertions made derogatory of private asylums before the Select Committee of the House of Commons in 1858, that we can only marvel. However, we shall merely refer to the report of the Commissioners, and add, that notwithstanding the carefully recounted items of suggested further improvements in the arrangements of several of the houses, the accounts of the Commissioners entirely bear out the favourable opinions we have been accustomed to express of the general character and management of our private asylums.

The recalcitrant licensed houses in the country, seven in number, are next noted, and the state of the lunatic hospital, Fort Pitt, Chatham, passed under review; and the instances in which insane soldiers have been set at large in the streets, in order to "raise the question as to the legal liability of the parish authorities to take charge of lunatic soldiers who might be now free in the parish in which Fort Pitt is situated," are animadverted upon. The question at issue is not yet definitively settled.

The condition of single private patients is largely entered upon by the Commissioners, and several painful illustrations are given to show the necessity which exists for a more effective supervision of these cases. The Commissioners report that—

"The general result of our experience of the system of treating the insane as single patients, strongly convinces us of the necessity for exercising, in all cases, the most careful supervision over them, both legislative and otherwise. Although in some instances there may be urgent reasons for giving this mode of treatment a trial, more especially in cases likely to be of short duration, it should ever be remembered that these are the cases in which medical and moral treatment are of the utmost importance, and that, if improvement does not take place within a limited period, much mischief may result from persevering in it. As a general rule, indeed, patients of this class are usually under much less advantageous circumstances, so far as the chances of recovery are concerned, than if placed in a well-conducted Asylum." (p. 69.)

The allowances of these patients are discussed, and the necessity of the Commissioners "ascertaining the amount of property to which insane patients are entitled, and the proper application of it to their use," is insisted upon.

The state of Single Pauper Patients is also discussed, and the Commissioners suggest:—

"1. That the Relieving Officer should be directed to regard such cases, not merely as paupers needing parochial relief, but as patients requiring medical treatment; that he should be instructed to give immediate notice of every new case to the Medical Officers; and that he should be made sensible of the necessity of affording a sufficient liberal allowance to all the insane and idiotic detained in the district, to whom also he should pay frequent visits and ascertain the nature of the accommodation and treatment, and whether the money granted is duly applied.

"2. That the Medical Officer should be desired when making his visits to give a larger amount of consideration to the wants of his charge, and not to rest satisfied with a mere examination into the personal condition of the patient when visited; that his inquiries should extend to the kind of accommodation provided, especially the sleeping room and bedding, the supervision and treatment, restraint, diet, and clothing, and whether the payment made by the relieving officer is sufficient.

"That his quarterly list should not contain merely an account of the patients visited at the end of the quarter, but should embrace all seen during the past three months.

"That he should more strictly comply with the statutory injunction in attesting that a patient is 'properly taken care of, and may properly remain out of an asylum.'

"3. That the clerk to the Board of Guardians be called upon to perform the duties required of him with punctuality and accuracy, both as respects the annual and quarterly returns." (p. 88.)

It is further suggested that "every Visitor of an Asylum, resident in an Union, and consequently an *ex-officio* Guardian, may, as such, be a most useful agent towards amending the condition of Pauper Patients boarded or sent to a Workhouse." His visits to the County Asylum would give him an amount of experience which might be most beneficially applied in the improvement of the state of pauper lunatics, and, the Commissioners say, "it seems to us that if any means could be adopted of introducing into each Union a member of the Committee of Visitors of the County Asylum, many advantages would arise out of the combination thus formed. To effect this object it would simply be necessary to add a certain number of names to the list of each Committee of Visitors. In many counties the addition would be of small amount; for instance, in Bucks seven, Cambridge nine, Derby nine, Chester ten, Dorset twelve." The advantages which the Commissioners conceive would arise from this arrangement are thus stated:—

"Such an arrangement would, we think, be followed by the best results, especially if in addition to it the Medical Officer of the district were entrusted with a larger amount of authority. It is not difficult to conceive that if a resident Magistrate were, as a member of the Committee of Visitors, to act in concert with the Medical Officer of the district, much substantial benefit and protection would be extended to this class of patients. The home treatment would be improved; removal whenever necessary would be more readily effected; and the information contained in the order and certificates would be more accurate; the highly objectionable practice of taking a patient for examination to a police court would be discontinued; and it is probable also that the obstacle raised to the transmission of a patient to an asylum owing to the payment of fees exacted by clerks to magistrates for drawing up the orders for admission, would be abolished." (p. 89.)

The particulars connected with the discharge from private asylums of the two lunatic murderers, James Moore and Dr. Pownall, are recorded, and in both instances the conduct of the medical superintendents of the asylums, in permitting these patients to go at large, is strongly condemned by the Commissioners.

Finally, the site and arrangements of the State Asylum for criminal lunatics, now in course of erection, are briefly described.

This asylum is situated at Broadmoor, on Bagshot Heath, distant about thirty-three miles from London. The land for the use of the asylum comprises about 290 acres, and is of undulating character, falling towards the south, and varying in elevation from 100 to 200 feet above the level of the sea. "The estate is bounded by plantations, principally of Scotch fir, and from the buildings fine and extensive views are commanded." The supply of water is abundant, and the natural facilities for land and house drainage good.

"The Asylum consists of a main building, and four separate blocks towards the north-east, north-west, south-east, and south-west, respectively, for Male Patients; and a detached building to the east for Females. The buildings are of three stories, and the style and elevations of a simple and pleasing character. The residence of the Medical Superintendent is conveniently situate midway between the Male and Female divisions."

It has been deemed sufficient for the present to provide accommodation for 400 males and 100 females. The buildings will readily admit of extension.

It is proposed that—

"The two wings of the main building should be appropriated each to 100 Male Patients of the ordinary class. Those of the better class as respects station of life and conduct will occupy the South-Western Block, the North-Western being assigned to working patients. The South-Eastern Block is intended to be used as the Male Infirmary, and the North-Eastern will be appropriated to the worst and most refractory cases of Male Patients. Each of the four Blocks is calculated to accommodate 50 patients. The Female Building for 100 patients will, as to kitchen, offices, and otherwise, be a separate Establishment. The Chapel for the Patients of both sexes is in the centre of the main building. The second-floors, throughout, are appropriated to sleeping accommodation."

On the 1st of January, 1860, the number of criminal lunatics amounted, according to the text of the report and a special table

### SUMMARY.

| | Number of Patients, 1st Jan., 1859. | | | | | | | Admissions during the Year 1859. | | | Discharges during the Year 1859. | | | | | | Deaths during the year 1859. | | | | | | | | |
|---|---|---|---|---|---|---|---|---|---|---|---|---|---|---|---|---|---|---|---|---|---|---|---|---|---|
| | Private. | | | Pauper. | | | Total Lunatics. | | | | Total Number. | | | Number Recovered. | | | Total Number. | | | From Suicide. Act committed in Asylum. | | | From Suicide. Act committed before Admission. | | |
| | M. | F. | Total. | M. | F. | Total. | | M. | F. | Total. | M. | F. | Total. | M. | F. | Total. | M. | F. | Total. | M. | F. | Tot. | M. | F. | Tot. |
| County and Borough Asylums, | 122 | 105 | 227 | 7129 | 8489 | 15618 | 15845 | 3074 | 3154 | 6228 | 1419 | 1510 | 2929 | 966 | 1154 | 2120 | 956 | 756 | 1712 | 5 | 2 | 7 | — | 1 | 1 |
| Hospitals ........................... | 1003 | 773 | 1776 | 108 | 108 | 216 | 1992 | 430 | 413 | 843 | 328 | 377 | 705 | 173 | 221 | 394 | 95 | 50 | 145 | 2 | — | 2 | — | — | — |
| Metropolitan Licensed Houses, | 663 | 624 | 1287 | 465 | 799 | 1264 | 2551 | 519 | 586 | 1105 | 605 | 821 | 1426 | 155 | 199 | 354 | 145 | 141 | 286 | 3 | 1 | 4 | — | — | — |
| Provincial Licensed Houses ... | 837 | 704 | 1541 | 469 | 455 | 924 | 2465 | 505 | 423 | 928 | 451 | 404 | 855 | 182 | 183 | 365 | 109 | 73 | 182 | 2 | 2 | 4 | 2 | — | 2 |
| | 2625 | 2206 | 4831 | 8171 | 9851 | 18022 | 22853 | 4528 | 4576 | 9104 | 2803 | 3112 | 5915 | 1476 | 1757 | 3233 | 1305 | 1020 | 2325 | 12 | 5 | 17 | 2 | 1 | 3 |

| | PATIENTS REMAINING, 1st JANUARY, 1860. | | | | | | | | | | | | | | | | | | | | |
|---|---|---|---|---|---|---|---|---|---|---|---|---|---|---|---|---|---|---|---|---|---|
| | Private | | | Pauper. | | | Total Lunatics. | Number deemed Curable. | | | Found Lunatic by Inquisition. | | | Criminals. | | | Chargeable to Counties or Boroughs. | | |
| | M. | F. | Total. | M. | F. | Total. | | M. | F. | Total. | M. | F. | Total. | M. | F. | Total. | M. | F. | Total. |
| County and Borough Asylums, | 121 | 106 | 227 | 7829 | 9376 | 17205 | 17432 | 867 | 1085 | 1952 | 10 | 3 | 13 | 263 | 97 | 360 | 728 | 886 | 1614 |
| Hospitals ........................... | 998 | 754 | 1752 | 120 | 113 | 233 | 1985 | 133 | 187 | 320 | 22 | 14 | 36 | 119 | 15 | 134 | — | — | — |
| Metropolitan Licensed Houses, | 703 | 639 | 1342 | 194 | 408 | 602 | 1944 | 91 | 162 | 253 | 69 | 58 | 127 | 20 | 3 | 23 | 17 | 28 | 45 |
| Provincial Licensed Houses ... | 874 | 732 | 1606 | 377 | 373 | 750 | 2356 | 174 | 187 | 361 | 76 | 41 | 117 | 174 | 46 | 220 | 49 | 37 | 86 |
| | 2696 | 2231 | 4927 | 8520 | 10270 | 18790 | 23717 | 1265 | 1621 | 2886 | 177 | 116 | 293 | 576 | 161 | 737 | 794 | 951 | 1745 |

Included in Total Lunatics.

in the appendix, to 731—viz., 571 males and 160 females. According to the statistical summary the number was 737—viz., 576 males and 161 females, They were thus distributed :—

| | Male. | Female. | Total. |
|---|---|---|---|
| Asylums, County and Borough, including Northampton Hospital . . . | 269 | 96 | 365 |
| Northern Hospital . . . . . | 113 | 15 | 128 |
| Metropolitan Licensed Houses . . | 20 | 3 | 23 |
| Fisherton House . . . . | 154 | 4 | 158 |
| Other Provincial Licensed Houses . . | 20 | 4 | 24 |

The appendix contains the statistical tables showing the movement and cost of patients in asylums, and sundry papers relative to licences and changes of proprietorship, the Chester Asylum, St. Luke's Hospital, Single Pauper Patients, and Criminal Lunatics.

In the statistical tables the two columns under which were entered deaths from "accidents or violence" have been omitted, "as all cases terminating fatally from accidents of every description entered under these heads, and unaccompanied by the necessary particulars in explanation, were calculated to produce erroneous impressions."

On the preceding page we give the statistical summary for 1859 of the movement of patients in asylums.

---

